# Peculiarities of Prion Diseases

**DOI:** 10.1371/journal.ppat.1004451

**Published:** 2014-11-20

**Authors:** Walker S. Jackson, Clemens Krost

**Affiliations:** German Center for Neurodegenerative Diseases (DZNE), Bonn, Germany; Washington University School of Medicine, United States of America

## Introduction

Prion diseases (PrDs) are transmissible and fatal neurodegenerative diseases naturally occurring in humans and animals, “mad cow” disease being the most infamous. Their development and propagation requires endogenous prion protein (PrP) and derives from the conversion of PrP to a misfolded form, which combines with other misfolded PrP molecules to form small nuclei (seeds). The seeds can then result in an exponential increase in additional misfolded PrP molecules, eventually accumulating into large aggregates. However, the physiological roles of normal and misfolded PrP, mechanisms of the conformational transition, and the associated nature of the infectious and neurotoxic agents still remain enigmatic. In this review, we address five questions regarding PrDs that we are frequently asked by laypeople and scientists new to the field.

## What Causes PrDs?

Understanding the mechanisms of PrDs will likely lead to interventions, but researchers and physicians are challenged by three fundamentally different forms—acquired, sporadic and familial—comprising a wide range of highly heterogeneous subtypes and phenotypes with various clinical and histopathological features [1]. Sadly, the terminology in this field is frustratingly confusing and, for example, the distinctions between infectious and acquired or spontaneous and sporadic PrDs are sometimes blurred. Infectious means that an individual's disease could be horizontally transmitted to another individual. Acquired means an individual became diseased because of infection. The distinction seems subtle but is important because one term refers to the cause of the disease while the other refers to a feature of the mainfested disease. Moreover, all PrDs are infectious, but not all PrDs are acquired. For example, familial PrDs are caused by a mutation, not by being infected, but are nonetheless infectious. Spontaneous means the disease was not triggered by an infection, whereas sporadic means there is no evidence that the diseased individual was infected or had a mutation. Familial PrDs begin spontaneously and the same is likely true for sporadic cases.

**Figure 1:**
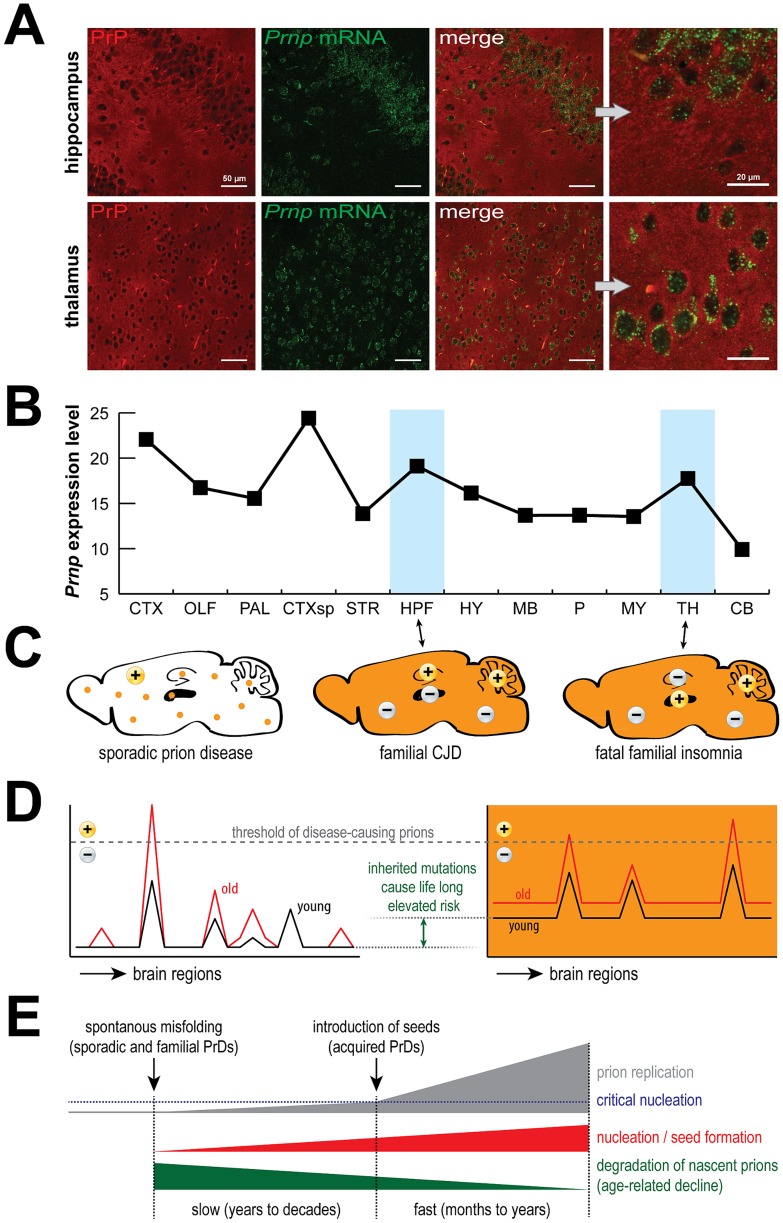
PrP expression, prions and disease. PrP is expressed by most cells (A) and is rather evenly distributed across the mouse brain (B). Surprisingly, PrDs may develop sporadically or be induced by mutations (C, orange color), but despite widespread expression, different regions are targeted in different familial PrDs (+) and the same is true for sporadic PrDs. Age-related effects likely permit disease-inducing prions to accumulate beyond a threshold that can be reversed in young but not old brains (D), which eventually leads to critical nucleation, followed by rapid replication and pathological changes (E). Data in (B) were obtained from the Allen Brain Atlas website (www.brain-map.org). Abbreviations: CTX, cortex; OLF, olfactory bulb; HPF, hippocampal formation; CTXsp, cortical subplate; STR, striatum; PAL, pallidum; TH, thalamus; HY, hypothalamus; MB, midbrain; P, pons; MY, medulla; CB, cerebellum. HPF is targeted in CJD mice, TH is targeted in FFI mice, and CB is targeted in both models.

Although acquired PrDs have immense notoriety, they account for less than 1% of human cases and are on the decline [2]. Since recent reviews have explored important questions of how prions enter the body and invade the brain [3], and mechanisms of familial prion diseases and the fascinating issue of selective vulnerability [4], [5], we focus here on sporadic human PrDs, representing the most common and yet least understood form. Surprisingly, even though they all involve PrP, cases can be phenotypically classified as sporadic Creutzfeldt-Jakob disease (sCJD), fatal insomnia, or protease-sensitive prionopathy [6]. While triggers for acquired and familial forms are clear (infections and mutations, respectively), the trigger for sporadic forms is murky, though there are some ideas. Considering the low incidence and the typically advanced age at onset, it seems likely that a certain threshold of disease-causing prions has to be exceeded before pathogenesis begins [7]. Since sporadic PrDs occasionally occur in adolescents [8], factors other than age, such as diet, environment, or genetics, may be involved. A transient perturbation of a microenvironment (inflammation, stress from dysregulated neural activity, deprivation of critical nutrients, impairment of waste removal, etc.) could induce wild-type PrP to spontaneously misfold into a prion conformation or impair its degradation, or permit the nucleation of prion seeds. Alternatively, a surprisingly large number of adult human neurons are aneuploid [9], and since increased expression of wild-type PrP can induce PrDs [10], increased PrP gene copies (and thus expression) could trigger the disease process. It is also conceivable that somatic mutations induce PrP to spontaneously misfold into low (non-toxic) levels of prions, and sometimes this process spirals out of control. These initial events then trigger the beginning of PrDs that propagate through the brain when accompanied by age-related changes of proteostasis, regardless of the initial cause. Notably, this is likely true for familial PrDs, too, which is remarkable since the disease-inducing mutant proteins are present throughout life, and yet emerge only after decades of expression. This implies that prions [7], and PrP itself [11], are effectively cleared from the brain by a mechanism that diminishes with aging. Just as the body usually removes cancers quite efficiently, the brain might routinely eliminate low levels of prions until adverse conditions give rise to a critical mass of prions, causing a lethal chain reaction (Figure 1). Regardless of how it begins, once a PrD is established it will progress rapidly, analogous to the instantaneous crystallization of a supersaturated salt solution upon addition of a minuscule crystal (seed).

## How Is It Known That Sporadic Prion Diseases Are Not Actually Acquired?

Two lines of reasoning support this conclusion. First, there are straightforward explanations of how this can happen. Recent experiments demonstrate that mutant forms of PrP do not require an infection to trigger the disease process [4], [5]. We speculate this is because the mutations either induce PrP to misfold into a prion conformation or impair its degradation, and the same probably happens for sporadic forms except that perturbation of the microenvironment orchestrates PrP's misfolding (see above).

Second, there are subtle but important differences between PrDs that can provide additional clues of an infection. Clinically acquired (iatrogenic) PrDs are typically identified by the correlation of a neuropathologically confirmed PrD following certain medical procedures, sometimes experimentally proven to be the cause [12]. Variant sCJD, caused by consumption of cattle with PrD (the infamous “mad cow” disease), was highly clustered geographically, often affecting young adults, and disease-related PrP aggregates were present in lymphoid tissue, a feature generally absent from other human PrDs [13]. In contrast, sporadic PrDs occur rather evenly (and sparsely) distributed across the globe, typically in people older than 50 years, without any telling epidemiological signals of localized clusters that would be indicative of infection. It is impossible to know with complete certainty that a sporadic PrD case is not actually caused by infection, but there is good reason to think not.

## How Can Pathological Processes of Sporadic PrDs Be Studied?

Human PrDs are intensively studied with respect to etiological, clinical, neuropathological, biochemical, molecular biological, and many other features. Nonetheless, this knowledge can be greatly enhanced using creative experimental models to reveal initiating processes. In addition to the widely used mouse models of acquired PrDs, which represent the human forms extraordinarily well, numerous transgenic mouse lines were generated to model familial PrDs [4], [5], [14]. Though they are not perfect, these models are well defined in terms of disease onset and progression, neuropathological and clinical features, making them quite useful to study familial PrDs. In contrast, modeling sporadic PrDs is extremely difficult without knowledge and use of a disease-initiating trigger. Nevertheless, two approaches are thought to reproduce aspects of these very rare diseases.

The first used a highly sensitive amplification technique to detect prions in brain extracts of uninfected, healthy wild-type mice [15]. However, it was concluded that the prions were generated in the in vitro reaction, rather than the brain, and therefore, in a sense, model an acquired disease when introduced back into an intact brain. In the second, mice genetically engineered to over-express wild-type PrP (from bank voles) spontaneously developed prion infectivity and disease amazingly reminiscent of conventional (acquired) prion models [10]. Is this a model of sporadic PrDs? The ideal model would include a population of seemingly similar individuals, where most do not but some do get sick, and none are infected or carry abnormal *Prnp* gene dosages or sequences. Such a model does not exist, though it would be invaluable for studying disease triggers. Therefore, despite enormous efforts and much progress, additional models of all forms of prion diseases, especially sporadic forms, are needed to reveal basic and specific mechanisms in humans.

## What Does “Prion-Like” Mean?

The term prion originally defined disease-inducing proteins that are infectious, but the definition is evolving in at least two new directions. In one direction, prion relates to proteins that naturally assemble into nuclear or cytoplasmic foci, often via prion domains. Prion assemblies were reported to provide positive biological functions in yeast [16]. Long used in the yeast prion field, the term “prion-like” caught the attention of an even wider audience with the report that a sea-slug protein carrying a domain similar to that in a yeast prion could assemble into a “prion-like” state that was beneficial for memory [17]. These and other works inspired the development of an algorithm to identify additional “prion-like” yeast proteins that performed new functions in a self-templating aggregated state [18]. The usage continued to spread (pun intended!) when this algorithm was applied to the mammalian genome and amino-acid stretches similar to those found in yeast prions were identified in self-assembling proteins that also tend to accumulate into pathological aggregates [19]. However, it should be recognized that the parameters of this algorithm were based on known yeast prion domains, which tend to have low complexity and a strong enrichment for glutamines and asparagines. Although mammalian PrP can be contorted into highly infectious prions, and it bears a low complexity region (i.e., repeated sequence), it lacks enrichment of glutamines and asparagines and, indeed, does not score highly as a prion with this algorithm. Importantly, other mammalian proteins appear to behave as prions in cancer [20] and inflammation [21], [22], suggesting there may be more “prion-like” mammalian proteins to discover.

The term “prion-like” is also evolving to propose mechanisms for spreading of neurodegenerative disease-related protein aggregates (such as those observed in Alzheimer, Huntington, and Parkinson [PD] diseases, to name a few) across the brain as diseases progress. Some quite provocative experiments have demonstrated that aggregated forms of neurodegenerative disease-related proteins, when extracted from brains of diseased humans or transgenic mice and injected into the brains of otherwise healthy mice, induced the formation of similar protein aggregates [23], [24], and sometimes similar processes were associated with toxicity [25]. This concept was invigorated by results of an experimental therapy for PD that involved grafting of fetal tissue into patients' brains. Several years later, the brains were examined histologically and the observation of PD-related aggregates in the grafted tissues [26] led many authors to suggest that the aggregates are spreading within the brain via a “prion-like” mechanism. These and similar observations have been extrapolated to suggest that the diseases might spread between individuals in a “prion-like” mechanism, though arguments against this have been articulated [27]. Therefore, the term “prion-like” is rather loosely applied and, though it is functionally useful to aid in scientific discussions, molecules receiving this label should not be assumed to be readily infectious between individuals.

## Are There Any Cures for Prion Diseases?

Unfortunately, there are currently no cures for any neurodegenerative disease. But a number of groups have worked tirelessly to uncover therapies targeted for PrDs and some recent reports offer hope. One approach is to eliminate PrP, the substrate necessary to maintain prion diseases. Suppression of PrP expression using transgenic tools was quite effective [28], and with the ongoing development of new technologies for gene delivery, there is hope this will be a viable strategy in the future.

An alternative, perhaps complementary approach would be to chemically interfere with disease progression. Many compounds have been identified that show great promise, but for brevity we describe just one example, anle138b [29]. When administered to mice 120 days after prion infection, a time when mice are clinically affected, it extended survival from approximately 160 days to approximately 220 days, representing approximately 7% of normal lifespan. The question then becomes if the treatment was applied to humans at a similar stage of disease and was equally effective, would it extend lifespan 60 days or 7% of expected lifespan (5 to 6 years for developed nations)? Amazingly, anle138b also modified disease progression in a model of familial PD and two models of chemically induced PD [29]. It is tempting to speculate that both diseases involve a common, prion-like mechanism that was targeted by anle138b. The truly remarkable point is that this molecule was discovered from studies of PrDs, and it highlights the fact that, although these diseases are rare in humans, studies of PrD mechanisms and interventions are certainly worthwhile.
